# Dr. Jackson’s Granula

**Published:** 1876-12

**Authors:** 


### *DR. JACKSON'S GRANULA.*

Messrs. Austin, Jackson & Co., proprietors of the famous Health Resort at Dansville, N. Y., known as " Our Home on the Hillside," are putting up in neat packages an article of Cooked Food which they call " Granula." It is made of choice wheat which passes through several cooking processes in order to fit it perfectly for digestion by feeble stomachs. From this excellent food—which contains all the nutriment of the best grain—it is not claimed that the silicious and woody hull is removed ; all harshness and all tendency to induce cathartic action, is, however, avoided by the semi-carbonization and subsequent powdering of the whole, by which the shells of the wheat are so reduced as to pass through the intestinal canal without lacerating it. No ordinary cooking process is competent to convert wheat meal or " Graham," or crushed wheat, or any crude grain, into food suitable for civilized man ; but the careful methods made use of in preparing this " Granula," result in a food which even weak digestive powers are able to assimilate. It is of a delicate light brown color, having a pleasant parched flavor, and is ready to eat after simply being stirred into milk or water and soaking for a short time. The Health Food Company are the agents for the sale of this valuable food.

